# Coronaviruses Are Abundant and Genetically Diverse in West and Central African Bats, including Viruses Closely Related to Human Coronaviruses

**DOI:** 10.3390/v15020337

**Published:** 2023-01-25

**Authors:** Dowbiss Meta Djomsi, Audrey Lacroix, Abdoul Karim Soumah, Eddy Kinganda Lusamaki, Asma Mesdour, Raisa Raulino, Amandine Esteban, Innocent Ndong Bass, Flaubert Auguste Mba Djonzo, Souana Goumou, Simon Pierre Ndimbo-Kimugu, Guy Lempu, Placide Mbala Kingebeni, Daniel Mukadi Bamuleka, Jacques Likofata, Jean-Jacques Muyembe Tamfum, Abdoulaye Toure, Eitel Mpoudi Ngole, Charles Kouanfack, Eric Delaporte, Alpha Kabinet Keita, Steve Ahuka-Mundeke, Ahidjo Ayouba, Martine Peeters

**Affiliations:** 1 Centre de Recherche sur les Maladies Emergentes et Réémergentes (CREMER), Yaounde P.O. Box 1857, Cameroon; 2 TransVIHMI, University of Montpellier, Institut de Recherche pour le Développement, INSERM, 34394 Montpellier, France; 3 Centre de Recherche et de Formation en Infectiologie de Guinée (CERFIG), Gamal Abdel Nasser University (UGANC), Conakry BP6629, Guinea; 4 National Institute of Biomedical Research (INRB), Kinshasa P.O. Box 1197, Democratic Republic of Congo; 5 Service de Microbiologie, Cliniques Universitaires de Kinshasa, Kinshasa P.O. Box 1197, Democratic Republic of Congo; 6 Institut National de Recherche Biomédicale (INRB), Kinshasa P.O. Box 1197, Democratic Republic of Congo; 7 Laboratoire Provincial de Mbandaka, Mbandaka, Democratic Republic of Congo; 8 Department of Public Health, Faculty of Health Sciences and Techniques, Gamal Abdel Nasser University (UGANC), Conakry P.O. Box 1147, Guinea

**Keywords:** bat, coronavirus, Africa, diversity, Sarbecovirus, *Rhinolophus*

## Abstract

Bats are at the origin of human coronaviruses, either directly or via an intermediate host. We tested swabs from 4597 bats (897 from the Democratic Republic of Congo (DRC), 2191 from Cameroon and 1509 from Guinea) with a broadly reactive PCR in the RdRp region. Coronaviruses were detected in 903 (19.6%) bats and in all species, with more than 25 individuals tested. The highest prevalence was observed in *Eidolon helvum* (239/733; 39.9%) and *Rhinolophus* sp. (306/899; 34.1%), followed by *Hipposideros* sp. (61/291; 20.9%). Frugivorous bats were predominantly infected with beta coronaviruses from the Nobecovirus subgenus (93.8%), in which at least 6 species/genus-specific subclades were observed. In contrast, insectivorous bats were infected with beta-coronaviruses from different subgenera (Nobecovirus (8.5%), Hibecovirus (32.8%), Merbecovirus (0.5%) and Sarbecovirus (57.6%)) and with a high diversity of alpha-coronaviruses. Overall, our study shows a high prevalence and genetic diversity of coronaviruses in bats and illustrates that *Rhinolophus* bats in Africa are infected at high levels with the Sarbecovirus subgenus, to which SARS-CoV-2 belongs. It is important to characterize in more detail the different coronavirus lineages from bats for their potential to infect human cells, their evolution and to study frequency and modes of contact between humans and bats in Africa.

## 1. Introduction

Emerging infectious diseases (EID) represent a major threat to global health. The majority of EIDs are the result of spillover events of pathogens from wildlife and the current COVID-19 pandemic with a new coronavirus, severe acute respiratory syndrome coronavirus, SARS-CoV-2, is a perfect illustration of the impact of zoonotic transmission. Since the first cases have been reported in China, almost 6.6 million people died and 630 million individuals became infected by mid october 2022 [1]. Most likely these numbers are even underestimated, because a high discordance has been observed between confirmed cases and seroprevalence in sub-Saharan Africa [2,3,4,5]. The presumed host reservoir for the current COVID-19 pandemic is insectivorous *Rhinolophus* bats in Asia, and a recent study suggests at least two zoonotic events with one lineage that became predominant [6].

To date, seven human coronaviruses (HCoVs) are known, including the recent SARS-CoV-2 virus [7,8]. They belong to the alpha (HCoV-229E and HCoV-NL63) and beta-CoV genera (HCoV-OC43, HCoV-HKU1, SARS-CoV-1 and 2, Middle East respiratory syndrome coronavirus (MERS-CoV)). Whereas HCoV-229E, HCoV-OC43, HCoV-HKU1 and HCoV-NL63 usually cause mild symptoms, such as common cold and/or diarrhea, SARS-CoV-1 and MERS-CoV are highly pathogenic, causing severe lower respiratory tract infection [7,8]. The newly identified SARS-CoV-2 is less pathogenic and more transmissible compared to SARS-CoV-1 and MERS-CoV but more pathogenic than the other HCoVs [7,8]. All seven HCoVs have a zoonotic origin; some have been directly transmitted to humans from bats, but for others, an intermediate host was required, such as camels for MERS-CoV and civets for SARS-CoV-1 [9,10,11,12,13,14,15]. For SARS-CoV-2, the role of an intermediate species has not yet been clarified [16,17,18]. However, all HCoVs have an evolutionary origin from bats, where viruses are well adapted and presumably non-pathogenic. The prevalence of coronaviruses is high in certain bat species, and a high genetic diversity with multiple subgenera of alpha and beta coronaviruses has been reported, thus providing multiple opportunities for spill-over events and the emergence of novel HCoVs [19].

It is thus important to be prepared for new outbreaks with coronaviruses. A major step in understanding the risk of zoonotic infections is to characterize pathogen diversity at the interface between humans and animals. Knowing the animal sources, extent of the animal reservoir (i.e., prevalence and geographic distribution) and the genetic diversity or evolutionary history of pathogens in wildlife is critical to predict risk for potential emergence or re-emergence of diseases. Bats are the most widely distributed terrestrial mammals in the world and constitute nearly 20% of mammalian biodiversity, with almost 1400 species now recognized [20]. Contacts between humans and bats are diverse and range from direct exposure to infected blood or tissues through hunting and butchering to indirect exposure to bat guano or fruit contaminated by their saliva, urine or feces [21,22]. A wide diversity of bat species are hunted for food or medicine, and bushmeat hunting is increasingly recognized as a major conservation threat for bats, primarily in Southeast Asia and West and Central Africa [21,23,24,25]. Given the frequent contact between bats and humans in Africa, it is important to document in detail the genetic diversity of CoVs in African bats, especially in the context of limited or under-equipped health facilities that could not allow early detection or recognition of new diseases. Although more than 26,000 bats have been tested in Africa and sequences close to HCoVs have been reported in bats from Africa, certain species and regions are still underrepresented [26]. Here, we report the prevalence and diversity of coronaviruses in bats sampled in West and West Central Africa.

## 2. Materials and Methods

### 2.1. Study Sites and Sample Collection

Samples were collected from free-ranging frugivorous and insectivorous bats in Guinea, Cameroon and the Democratic Republic of Congo (DRC) between November 2015 and February 2022, as previously described [27]. Briefly, bats were captured using mist nets or harp traps in roosting and foraging sites and released immediately after sampling. Venipuncture was done of the propatagial or brachial vein and blood drops were directly transferred onto Whatman 903 filter paper (GE-Healthcare, Feasterville-Trevose, PA, USA), which were air-dried and preserved individually in plastic bags containing silica desiccant and stored in a hermetic box as dried blood spots (DBS). DBS (a maximum of 2–3 weeks after collection) were subsequently stored frozen (−20 °C) until analysis. Rectal and/or oral swabs were collected and stored in 500 µL RNA-later (Ambion, Austin, TX, USA) in the field at ambient temperature for a maximum of two weeks and subsequently frozen in the laboratory. For each bat that was sampled, information on capture sites (GPS coordinates, ecological environment), capture method, morphology (body measurements, weight, color), sex, age class (adult, juvenile) and visual species identification were recorded. In Cameroon, adults were also classified into subadults and mature adults based on the presence of enlarged testes and/or distended cauda epididymis for males and the development and morphology of the mammary glands and thoracic (axillary) and pubic nipples for females.

### 2.2. Nucleic Acid Extraction and Screening for the Presence of Coronavirus RNA

Total DNA and RNA were extracted from swabs and feces using the NucliSENS EasyMAG platform (BioMérieux, Marcy-l’Etoile, France) or the Qiagen Viral RNA mini kit (Les Ulis, France). Briefly, for the Nuclisens method, 250 µL of sample was incubated with 2 mL of lysis buffer for 15 min and extraction was performed using the manufacturer’s instructions. The total nucleic acids were resuspended in 60 µL elution buffer. For the Qiagen method, nucleic acids were extracted from 250 µL of sample as per the manufacturer’s instructions. RNA was eluted in 60 µL elution buffer. Extracted RNAs were stored at −80 °C until use. For the detection of coronaviruses, cDNA was first synthesized from denatured RNA (70 °C for 10 min) using a Reverse Transcription System kit with random primers (Promega, Madison, WI, USA), following the manufacturer’s instructions. Broadly reactive degenerate primers were used to amplify a 440 base pair (bp) fragment in the highly conserved RdRp region by nested PCR [28]. Amplification conditions were slightly adapted, as previously described [29]. cDNA was amplified with the GoTaq Hot Start Master Mix PCR kit (Promega, Madison, WI, USA) to obtain a 620 bp fragment in the first round with 40 cycles of 92 °C for 30 s, 48 °C for 30 s and 72 °C for 50 s. Cycling conditions for the second round used a touch-down technique to reduce non-specific amplifications (10 cycles of 92 °C for 30 s, 53 °C for 30 s with −0.5 °C/cycle and 72 °C for 30 s) followed by 35 cycles of 92 °C for 30 s, 53 °C for 30 s and 72 °C for 30 s). The PCR products were visualized by agarose gel electrophoresis. PCR products were directly sequenced with a BigDye Terminator version 3.1 sequencing kit (Life Technologies, Courtaboeuf, France) on an Applied Biosystems 3500 Genetic Analyzer (Thermo Fisher Scientific, Foster City, CA, USA). Sequences from both strands were reconstituted using the SeqMan Pro tool from the package DNAStar v17.0.2 (Lasergene, Madison, WI, USA). The newly obtained RdRp sequences of coronaviruses were then analyzed with the R package MyCoV for rapid classification in subgenera [30]. The MyCoV package is available at https://github.com/dw974/MyCoV (accessed on 19 May 2022).

### 2.3. Phylogenetic Analyses

The new RdRp sequences were aligned with representatives of the different subgenera of alpha and beta coronaviruses and with sequences reported from bats in Africa from the same regions, when possible. Multiple sequence alignment (MSA) was obtained using MAFFT v7 (https://mafft.cbrc.jp/alignment/server/, accessed on 23 August 2022). The alignment was manually checked and end-trimmed to match the newly obtained RdRp sequences and to remove the PCR primer sequences. The final alignment was used for maximum likelihood (ML) phylogenetic analysis with GTR + F + I + 4Γ as the best-fit model of nucleotide substitution according to BIC and 1000 bootstrap resampling using the IQ-Tree server (http://iqtree.cibiv.univie.aC.at, accessed on 23 August 2022) [31,32]. Consensus trees were edited with FigTree v1.4.4 (http://tree.bio.ed.ac.uk/software/figtree/, accessed on 23 August 2022).

### 2.4. Molecular Confirmation of Bat Species

Species identification recorded in the field was molecularly confirmed for the majority of bats that were infected with a coronavirus using the corresponding DBS sample, as in our previous studies on viruses in bats [27,33,34]. In addition, at least one sample per sampling date, per capture method and per morphologic description at each site was confirmed. A 800 bp fragment of the mitochondrial cytochrome b (CytB) region was amplified using previously described primers to identify mammal species, including bats, Cytb-L14724 (forward) and Cytb-H15506 (reverse) [35]. To increase PCR specificity for certain bat species, the forward primer was replaced by a newly designed primer Cytb-L1 5′-ATG ACC AAC ATC CGA AAA TCN CAC-3′ or Cytb-L2 5′-ATY TCY TCM TGA TGA AAY TTY GGM T-3′ [34]. For a subset of samples, species identification recorded in the field was confirmed by amplifying a 386-bp mitochondrial DNA fragment of the 12S rRNA gene with primers 12S-L1091 and 12S-H1478 [36]. The PCR products were directly sequenced and reconstructed, as described above. Sequences were pasted in the NCBI BLAST web interface (https://blast.ncbi.nlm.nih.gov/Blast.cgi, accessed on 12 May 2022) to identify the most similar bat species. For samples with no or low similarity (<97%) hits with species in Genbank, a phylogenetic tree was constructed using maximum likelihood methods implemented in PhyML with reference sequences in order to obtain genus identification. Species identification was extrapolated for the remaining samples by combining molecular and field data. For certain insectivorous bats (from *Molossidae*, *Rhinolophidae*, *Hipposideridae* and *Nycteridae* families), identification was only possible at the genus level, mostly due to the lack of reference sequences in Genbank. For *Epomophorus gambiensis* and *Micropteropus pusillus,* species discrimination cannot be done by CytB sequences only, and morphologic details on the forearm and weight measurements were also used to discriminate the species, as previously described [34,37].

### 2.5. Statistical Analyses

To explore the impact of age, sex and reproductive stage of the collected samples on the detection of coronavirus RNA, we performed Kruskal–Wallis or Chi-2 tests. Significant results were considered for a *p*−value of <0.05.

## 3. Results

### 3.1. Bat Samples

**Figure 1:**
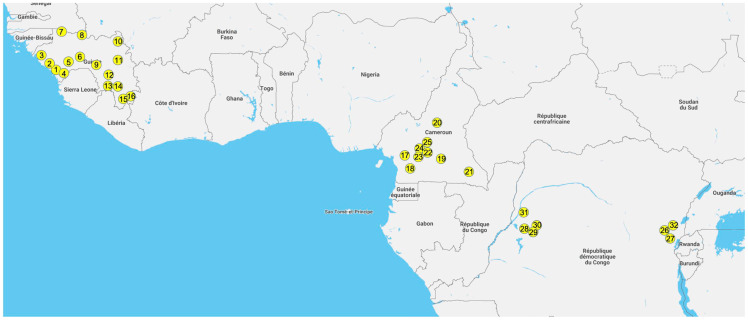
Collection sites of bat samples. Sites where bat samples were collected are highlighted with yellow circles. Sites are indicated with numbers as follows in the three different countries: Guinea (1, Conakry; 2, Boffa; 3, Boke; 4, Forecariah; 5, Kindia; 6, Mamou; 7, Koundara; 8, Mali; 9, Faranah; 10, Siguri; 11, Kankan; 12, Kissidougou; 13, Gueckedou; 14, Macenta; 15, Nzerekore; 16; Lola), Cameroon (17, Bipindi; 18, Campo; 19, Doumou Pierre; 20, Tibati; 21, Mambele; 22, Yaounde; 23, Mbankomo; 24, Nkolbisson; 25, Obala) and DRC (26, Beni; 27, Butembo; 28, Bikoro; 29, Iboko; 30, Ingende; 31, Mbandaka; 32, Mangina). Details on the number of samples per site are shown in Supplementary Table S1.

Swabs from a total of 4597 bats were analyzed, 897 from DRC, 2191 from Cameroon and 1509 from Guinea, including 319 bats from a previous pilot study [29]. The samples were collected at various sites across the 3 countries: 16 for Guinea, 9 for Cameroon, and 7 for DRC (Figure 1 and Supplementary Table S1). Bats were captured in different ecological environments: villages (15.5%), forest sites (20.2%), urban sites (17.3%), plantations (7.3%), caves (25.9%) and diverse other settings for the remainder. For a total of 1760 (38.2%) bats, species identification in the field was confirmed by sequence analysis. Details on bat families, genera and species analyzed in this study are shown in Supplementary Table S2. Overall, we analyzed a total of 2986 (65%) frugivorous bats from at least 12 species. Among them, the predominant species were *Rousettus aegyptiacus* (*n* = 791), *Epomophorus* sp. (*n* = 737) and *Eidolon helvum* (*n* = 733), followed by *Epomops* sp. (*n* = 258), *Micropteropus pusillus* (*n* = 165), *Hypsignathus monstrosus* (*n* = 103), *Myonycteris torquata* (*n* = 91) and *Lissonycteris angolensis* (*n* = 65). Among the 1611 (35%) insectivorous bats, at least 7 different families were represented; the highest number of bats were from the *Rhinolophus* genus (*n* = 899), followed by species from the *Mollosidae* (*n* = 399) and *Hipposideridae* (*n* = 291) families. Marginal numbers of samples, less than 25, were obtained for 4 frugivorous bat species (*Casinycteris arginnis* (*n* = 24), *Megaloglossus woermanni* (*n* = 15), *Nanonycteris* sp. (*n* = 3) and *Scotonycteris* sp. (*n* = 1)) and 5 insectivorous bat species (*Coleura afra* (*n* = 1), *Miniopterus* sp. (*n* = 9), *Nycteris* sp. (*n* = 5), *Myotis* sp. (*n* = 4) and *Scotophilus* sp. (*n* = 3)), which were often only captured in a single country. Among the predominant species, the majority were captured in the three countries, as is the case for *E. helvum*, *Hipposideros* genus and representatives of the *Mollossidae* family. *R. aegyptiacus* was only captured in Guinea and Cameroon. *Epomophorus* species were only captured in DRC and Guinea. In addition, in DRC in the East, *E. labiatus* was captured versus *E. gambiensis* in Western DRC and Guinea. Concerning the *Epomops* genus, two species were captured: *E. franqueti* in DRC and Cameroon and *E. buettikoferi* in Guinea. *Rhinolophus* bats were only captured in Cameroon and Guinea.

Overall, 2104 (45.8%) were male and 2480 (53.9%) were female bats; for 13 (0.3%) bats, the sex was not recorded. Only 391 (8.5%) were juvenile bats, 4139 (90.0%) were adults, and for 67 (1.5%), the age class was not available.

### 3.2. Detection and Genetic Diversity of Coronaviruses

**Figure 2:**
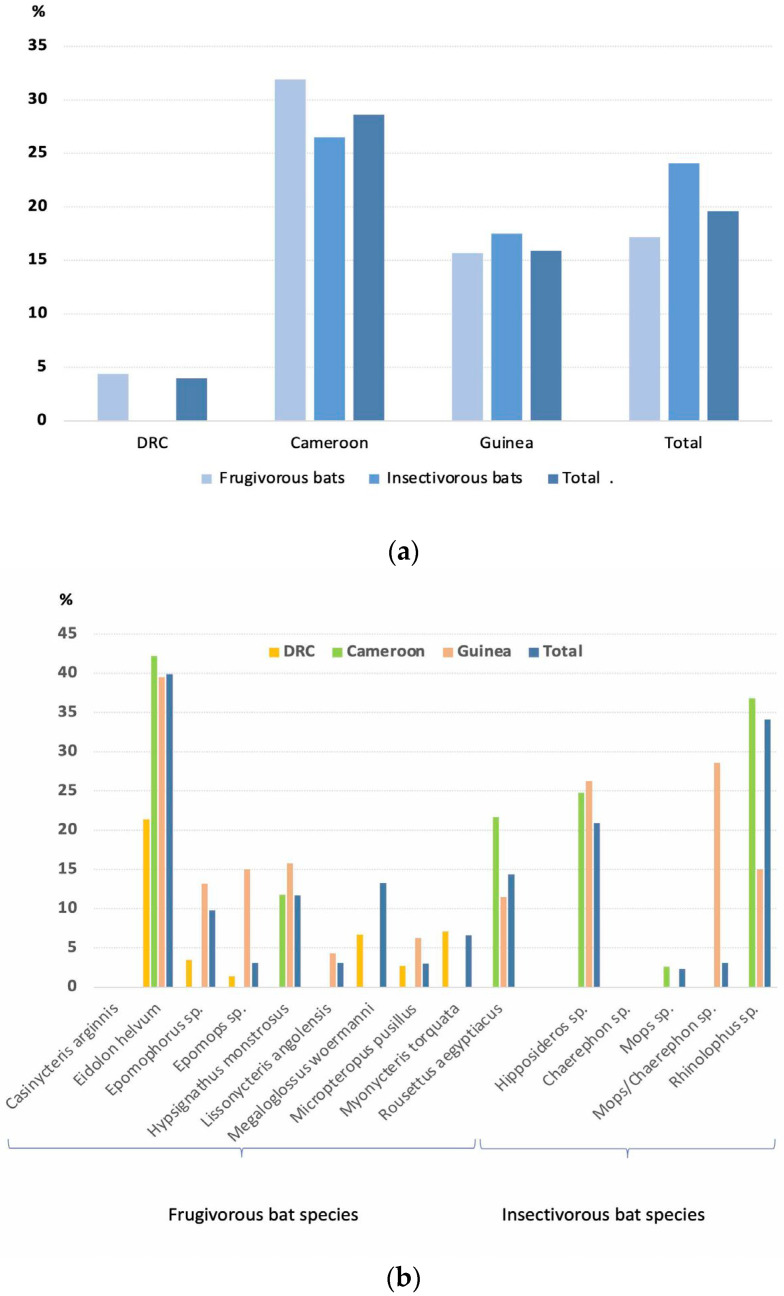
Number and percentages of bat genus/species positive for coronaviruses per country (**a**) and per species for which sufficient samples have been tested (**b**). Details are shown in Supplementary Table S2.

Among the 4597 bats tested by RT–PCR for the presence of coronavirus RNA, a total of 903 (19.6%) tested positive: 515/2986 (17.2%) of the frugivorous bats and 388/1611 (24.1%) of the insectivorous bats. CoVs were identified in all species, except *Casinycteris argynnis, E. buettikoferi* and *Scotonycteris bergmansi,* for which low numbers were tested, 24, 3 and 1, respectively. Positivity rates varied per species and were highest in *E. helvum* (29/733 (39.9%)), *Rhinolophus* sp. (306/899 (34.1%) and *Hipposideros* sp. (61/291 (20.9%)) for which large numbers of samples were collected. Intermediate rates were observed in *R. aegyptiacus* (114/791 (14.4%), *H. monstrosus* (12/103 (11.7%)) and *Epomophorus* sp. (72/737 (9.8%)) and lowest rates were seen in *M. torquata* (6/91 (6.6%)), *Epomops* sp. (8/258 (3.1%)), *L. angolensis* (2/65 (3.1%)), *M. pusillus* (5/165 (3.0%)) and in bats from the insectivorous *Mollossidae* family (10/399 (2.5%)). For the other species, sample numbers were low and detection rates were most likely not representative. Figure 2 shows percentages of frugivorous and insectivorous bats that were positive per country (Figure 2a) and per species (Figure 2b), and details are shown in Supplementary Table S2.

**Figure 3:**
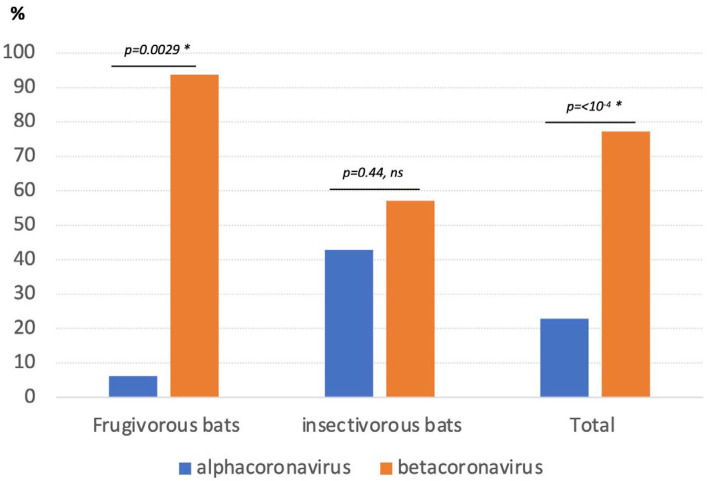
Proportion (%) of alpha and beta coronaviruses per type of bat. Details are shown in Supplementary Table S3. ns: non-significant χ^2^ test (*p*−value > 0.05); * significant χ^2^ test (*p*−value < 0.05).

Among the 903 PCR-positive samples, 768 (85.2%) were sequenced and revealed an overall predominance of beta-CoVs, 77.2% (593/768) (Supplementary Table S3). Frugivorous bats were predominantly infected with beta-CoVs, which represented 93.8% (395/421) of the sequences versus 57.1% (198/347) for insectivorous bats (Figure 3). Whereas alpha-CoVs were only marginally detected in the majority of frugivorous bat species tested, 21.6% (21/97) sequences from *R. aegyptiacus* were alpha-CoVs.

**Figure 4:**
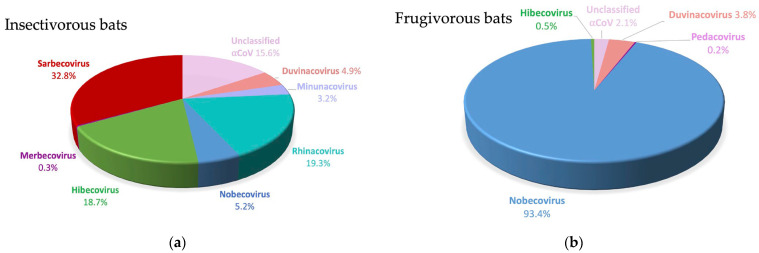
Proportion (%) of coronavirus subgenus sequences detected in positive insectivorous bats (**a**) and in frugivorous bats (**b**). The colors are consistent with the subgenus colors in Figure 5. Details are shown in Table 1.

As shown in Table 1 and Figure 4, representatives of all subgenera, except Embecovirus, were observed among the beta-CoVs. The vast majority were Nobecoviruses (69.3%), followed by Sarbecoviruses (19.2%), Hibecoviruses (9.6%) and a single sequence from a *Nycteris* bat in Guinea was classified as a Merbecovirus. Sarbecoviruses were only detected in *Rhinolophus* species in Cameroon and Guinea. Hibecoviruses were predominantly detected in *Hipposideros* bats from Cameroon and Guinea, but also in *Rhinolophus* bats in Cameroon captured in the same cave. Nobecoviruses were identified in all positive frugivorous species in a minority of *Hipposideros* and *Rhinolophus* species. All beta-CoVs observed in the *Molossidae* family belonged to the Nobecovirus subgenus. A wide diversity of alpha-CoVs was also observed, with representatives of Duvinacoviruses (*n* = 17), Minunacoviruses (*n* = 11), Rhinacoviruses (*n* = 67), sequences that are close to Pedacoviruses (*n* = 1) and Decacoviruses (*n* = 16) and 63 sequences that do not belong to currently described subgenera. The Duvinacovirus subgenus also harbors the HCoV-229E strain, which causes mild symptoms in humans across the world [38]. Figure 4 illustrates the higher diversity of subgenera observed in insectivorous versus frugivorous bats, i.e., 10 versus 4, respectively.

**Figure 5:**
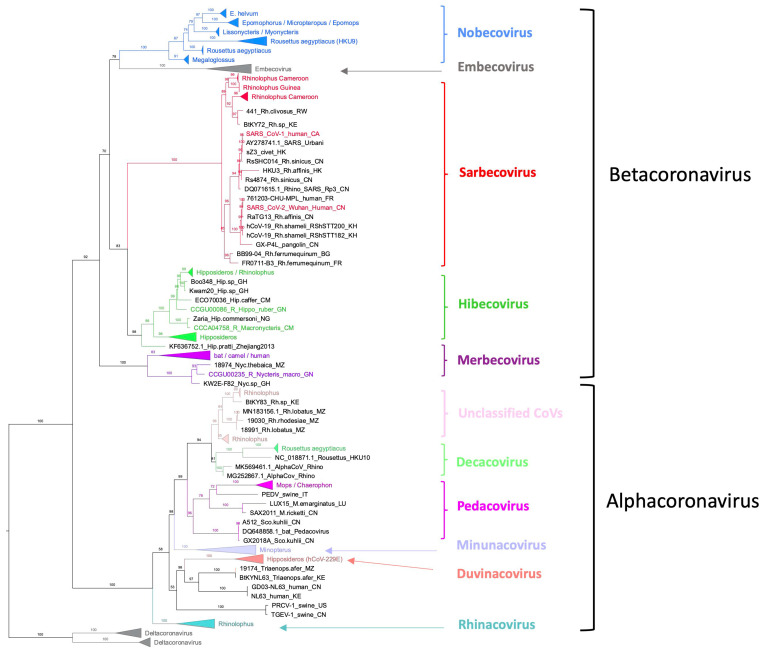
Phylogenetic tree with all new CoV sequences in the RNA-dependent RNA-polymerase (RdRp) partial nucleotide sequences (768 unambiguously aligned base pairs from bats obtained in this study). Phylogenetic analysis was performed as described in the methods and edited with increasing nodes and midpoint rooting in FigTree. Sequences in black and gray refer to reference sequences from the different CoV genera and sub-genera. Subgenera, including new CoV sequences from this study, are highlighted in color. The number of new sequences included in the different subgenera corresponds to the numbers indicated in Table 1 as follows: 255 Nobecoviruses (*Eidolon* cluster), 68 Nobecoviruses (*Epomophorus* cluster), 8 Nobecoviruses (*Lissonycteris* cluster), 69 Nobecoviruses (*Rousettus* cluster with HKU9), 9 Nobecoviruses (*Rousettus* cluster), 2 Nobecoviruses (*Megaloglossus* cluster), 67 Hibecoviruses, 1 Merbecovirus, 114, Sarbecoviruses, 63 unclassified alpha-CoVs, 16 Decacoviruses, 17 Duvinacoviruses, 11 Minunacoviruses, 1 Pedacovirus, 67 Rhinacoviruses. Details on reference sequences and new sequences are provided in Supplementary Tables S5 and S6, respectively.

Phylogenetic analysis of all newly identified sequences with references from the different subgenera and from other studies in Africa showed a high diversity in the Nobecovirus subgenus, with at least 6 different mostly species-specific subclades (Table 1, Figure 5). One major clade contained almost exclusively sequences amplified in *Eidolon* in Guinea, Cameroon and DRC and other previously published sequences from *E. helvum* bats from other countries in East, West and Central Africa. Among the 236 beta-CoVs identified in *E. helvum* bats in our study, 233 fell into this clade. A second clade contains mainly sequences from *Epomophorus* and *Micropteropus* bats, as well as from all regions confounded together with previously published sequences from these species. Sequences obtained from the two *Epomophorus* subspecies fell into the same clade. Sequences obtained from *Epomops franqueti* from DRC and *Epomops buettikoferi* bats from Guinea also fell within this clade. A third clade comprises sequences from *Lissonycteris* and *Myonycteris* bats from Guinea and DRC, respectively. Coronaviruses from *Megaloglossus* bats also formed a separate clade in the Nobecovirus subgenus. *Rousettus aegyptiacus* was infected with two species-specific Nobecovirus clades, one that included the HKU9 strain obtained from a *Rousettus* bat in Guangdong province in China [39] and a second clade with lower numbers of sequences from *R. aegyptiacus* bats from Guinea and Cameroon. Additionally, among the Hibecoviruses, a wide diversity was seen among the sequences obtained from the *Hipposideros* species. Sarbecoviruses were identified in *Rhinolophus* bats in Cameroon and Guinea.

**Figure 6:**
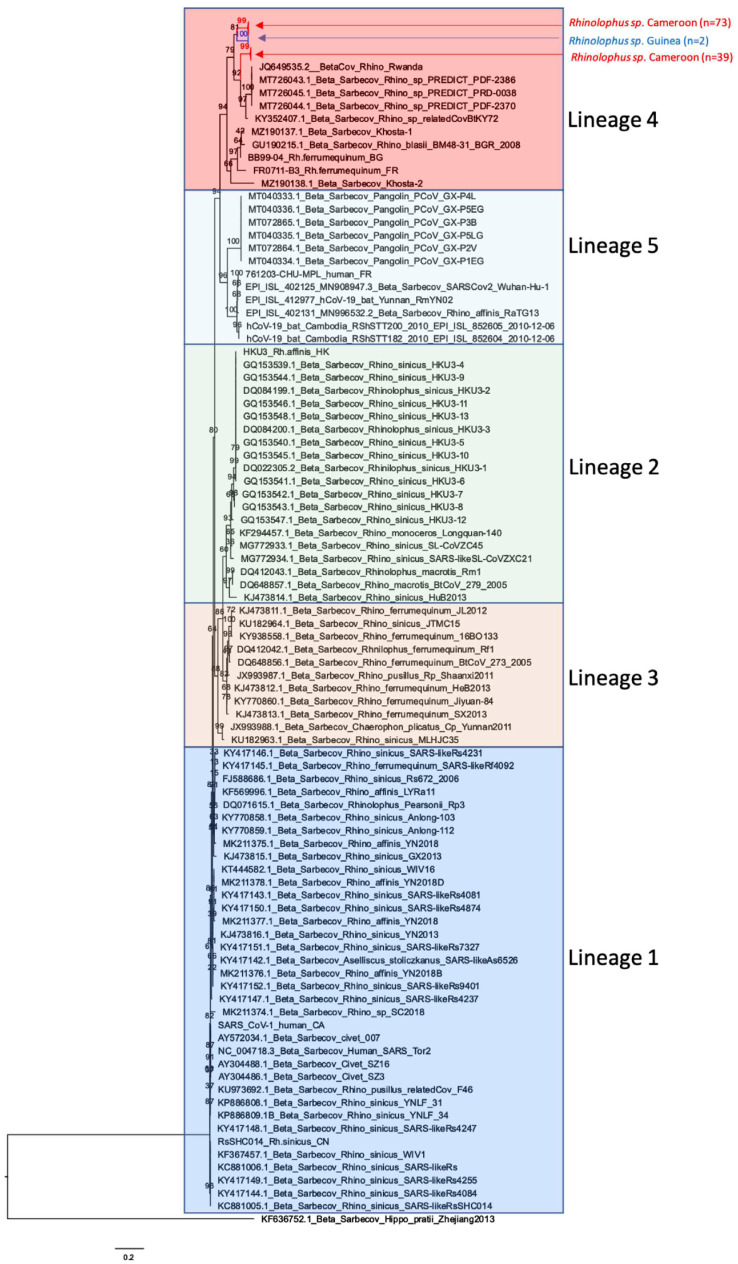
Phylogenetic tree with all new Sarbecovirus sequences detected from bats in this study and reference strains representing the 5 Sarbecovirus lineages previously described [40]. The new African Sarbecovirus sequences fall into the previously identified “lineage 4” containing strains from African and European *Rhinolophidae*. The sequences from Cameroon are highlighted in red and the sequences from Guinea in blue.

Among the Sarbecoviruses, two major clades were identified: one with sequences from Asia and the other with sequences from Africa and Europe. The Asian clade was further subdivided into four different lineages (i.e. lineage 1, 2, 3 and 5). The new African Sarbecovirus sequences fell into the previously identified “lineage 4,” containing strains from African and European *Rhinolophidae*, but the sequences from Cameroon and Guinea each fell into separate clusters within the cluster of strains from African bats (Figure 6) [40]. Moreover, the Sarbecoviruses amplified in *Rhinolophus* bats from Cameroon formed two separate clusters related to the year of collection. All new Sarbecovirus sequences from West and West Central Africa were different from those observed in bats from East Africa.

In general, each bat species/genus was infected with a predominant alpha- and/or beta-CoV variant independent of their geographic origin, but co-circulation with variants from other CoV subgenera or subclades from the same subgenus was also observed. For example, *Eidolon helvum* bats from Guinea, Cameroon and DRC were infected with the same Nobecovirus subclade, but a small minority (3/236 sequences) fell in the subclade dominated by *Epomophorus* and *Micropteropus* species. On the other hand, *Rhinolophus* bats were infected with a wide diversity of alpha- and beta-CoVs; i.e., at least 3 different subgenera of alpha-CoVs and 3 subgenera of beta-CoVs, including 3 Nobecovirus subclades (Table 1, Figure 5). *R. aegyptiacus* bats were also infected with a wide diversity of CoVs, at least 3 Nobecovirus subclades, Hibecoviruses and two alpha CoV subgenera.

### 3.3. Factors Associated with Detection of Coronaviruses in Bats

We compared whether age or sex could play a role in coronavirus positivity using PCR. Overall, among all bat samples tested, 18.0% (16.5–19.5%; 447/2480) of female and 20.9% (19.1–22.6%; 439/2104) of male bats were positive for coronaviral RNA (Table 2). For frugivorous bats, there was a trend of lower positivity in female bats, 14.7% (13–16.4%) versus 19.4% (17.3–21.5%) (Chi-2; *p* = 0.0007). This was not homogenous among species; for example, equal proportions of positivity were seen for *Epomophorus* sp. and *Rousettus aegyptiacus* bats, and a higher prevalence was seen in male bats for *Epomops* sp. and *Eidolon helvum* (Chi-2; *p* = 0.0197) (Table 2). In insectivorous bats, no overall difference in positivity was seen between female and male bats (Chi-2; *p* = 0.57), but the results differed according to species and genus, as shown in Table 2.

Prevalence was higher in juvenile bats; i.e., 34% (29.3–38.7%) versus 18.7% (17.5–19.9%) in adult bats (Chi-2; *p* < 0.0001) (Table 3). This trend was clearly confirmed for frugivorous bats (Chi-2; *p* < 0.0001) and for the majority of species for which sufficient samples from both age categories were tested, i.e., *Eidolon helvum* (Chi-2; *p* < 0.0001), *R. aegyptiacus* (Chi-2; *p* = 0.0001) and *Rhinolophus* sp. (Chi-2; *p* < 0.0001), except for *Epomophorus* bats, but the number of juvenile bats tested for this species was low. For insectivorous bats, equal detection rates were observed for juvenile and adult bats, but overall, a low number of juvenile bats was tested. In Cameroon, age determination of bats differentiated between adults and subadults. Supplementary Table S4 shows the percentage of PCR-positive bats for the three different age categories in this country and clearly illustrates that among adults, the prevalence was higher in subadults, i.e., 40.6% (36.0–44.2%) versus 18.9% (16.6–21.3%) in mature adults. Positivity rates in subadults were close to those observed in juvenile bats. This trend was observed in frugivorous and insectivorous bats and for all species with sufficient samples tested in the different age categories, i.e., *Eidolon helvum*, *Rousettus aegyptiacus* and *Rhinolophus* sp.

We also looked more in detail at whether the prevalence of coronaviruses differed in adult females according to the reproductive cycle, i.e., gestation or lactation. We observed no significant difference for gestating bats, 23.4% versus 21.2% (Chi-2; *p* = 0.09), but the detection rate of coronaviruses was lower in lactating females (11.8% (4.9–18.6%)) than in non-lactating bats (22.7% (18.6–26.7%)) (Chi-2; *p* = 0.035) (Table 4).

## 4. Discussion

We conducted a large survey to study the prevalence and genetic diversity of coronaviruses in almost 4600 bats from West and West Central Africa and provide significant new information on coronaviruses in bats from Guinea, West Africa and on species that were under-sampled in previous surveys in Cameroon and DRC, such as *Epomophorus* species in DRC or *Hypsignatus monstrosus*, *Mops* and *Rhinolophus* species in Cameroon [38,41]. Overall, we report a high prevalence of coronaviruses, with 19.6% of the bats that were infected. Prevalence can vary among species, but coronaviruses have been detected in almost all species for which sufficient samples have been tested. A prevalence above 30% was observed in the widely present frugivorous *Eidolon helvum* but also in *Rhinolophus* bats, for which high numbers have been tested. We also identified a wide diversity of coronaviruses, with representatives of almost all known subgenera of alpha- and beta-CoVs and identified a subset of strains that cannot be classified in the alpha-CoV genus. We detected bat CoVs (*n* = 199) that belong to 3 subgenera that also harbor HCoV strains known to be pathogenic for humans, i.e., Sarbecoviruses, Merbecoviruses and Duvinacoviruses. This is also the first study that shows that Sarbecoviruses are widely present in *Rhinolophus* bats in Africa, representing 114 of the 275 sequences (41.4%) retrieved from the 306 positive *Rhinolophus* bats that have been detected in this bat genus in our study.

As previously reported, we observed host specificity at the genus and/or species levels [26]. Nobecoviruses are largely predominant in frugivorous bats, with six major subclades that are associated with species or genus, as previously reported [26]. Nevertheless, some of these frugivorous species are also sporadically infected with Nobecoviruses from another subclade. We also show for the first time that *Hypsignathus monstrosus* are infected with Nobecoviruses, and mainly with the *Eidolon helvum*-specific clade, most likely because they roost in the same fruit trees, as is the case in Yaoundé, Cameroon, where the majority of them have been sampled and where a large colony of *Eidolon helvum* bats is present. Representatives of the Hibecovirus subgenus have been identified mainly in *Hipposideros* bats but also in a significant proportion of *Rhinolophus* bats in Cameroon, especially in those that share habitats in a cave located in the southwest, thus confirming host switching of coronaviruses among bat species that share habitats, as previously reported [19].

Sarbecoviruses are widespread in *Rhinolophus* bats in Asia and Europe and phylogeographic clustering has been reported in Asian *Rhinolophus* bats, which have been largely studied because Chinese *Rhinolophidae* are the natural reservoir for Sarbecoviruses [42,43,44]. Five subclades or lineages have been described for Sarbecoviruses by Yu et al. [43], with lineage 1 including SARS-CoV-1. Bat Sarbecovirus strains from Europe and Africa have been classified as lineage 4 and SARS-CoV-2 and other related viruses from *Rhinolophus* bats and pangolins from Asia fall into lineage 5 [40,45,46,47,48,49,50]. The Sarbecoviruses that we observed in *Rhinolophus* bats from Cameroon and Guinea fall into lineage 4 but form separate clusters with the other Sarbecoviruses identified in *Rhinolophus* bats from East Africa (Rwanda, Kenya and Uganda), thus illustrating phylogeographic clustering of African Sarbecoviruses. Nevertheless, Sarbecoviruses from Asian lineages 1 and 5 co-circulate in the same geographic area and are at the origin of SARS-CoV-1 and 2, respectively. This highlights that more sampling has to be done in *Rhinolophidae* in Asia, but also in Africa to examine further genetic diversity, geographic clustering and co-circulation of Sarbecovirus lineages. More studies are also needed to confirm whether all African strains are unable to utilize human ACE2 and are not able to infect human cells, as shown by in vitro studies [40]. However, it cannot be excluded whether the Sarbecoviruses from Africa use a different receptor. The colony of *Rhinolophus* bats infected at high rates (34%) with Sarbecoviruses has been identified in Cameroon in a cave that is frequently used by hunters as shelter for protection during bad weather conditions (rain) and where *Rousettus aegyptiacus* bats are hunted during the whole year for consumption (Ndong Bass, personal observation), which implies thus that humans might be in contact with guano (bat feces) dropped on the cave’s floor or with air saturated with contaminated aerosols. Our exploratory study on knowledge, attitudes and practices in Southern Cameroon showed that surrounding villages did not exploit bat guano as fertilizer [22], in contrast to what was reported, for example, in Zimbabwe [51].

Additionally, for alpha-CoVs, species-specific clades are seen; for example, Rhinacovirus and a separate clade of unclassified viruses were predominantly observed in *Rhinolophus* bats and Duvinacoviruses in *Hipposideros* bats. Nevertheless, *Rhinolophus*, *Hipposideros* and *Rousettus aegyptiacus* bats are infected with at least 3 to 6 different subgenera of alpha- and beta-CoVs, illustrating host switching most likely because they roost together in the same caves. Among the frugivorous bats, only *R. aegyptiacus* is infected to some extent with alphacoronaviruses, representing around 20% of the infections in this species. The majority belong to the Decacovirus subgenus, which includes the HKU10 strain identified in a *Rousettus* bat from China [52].

We also confirmed that juvenile and subadult bats are infected at higher rates than adult bats, as previously reported [19,53]. This was observed in all species for which sufficient juvenile and/or subadults have been included. We observed a trend in which males are more infected than females, especially in frugivorous bats, but this was heterogeneous according to the different species and could be due to sample bias. In our study, the prevalence of coronavirus infection was not different between gestating and non-gestating adult females, but in contrast to other reports, we observed lower rates in lactating females [54,55,56]. This difference could be due to different sample numbers or the predominance of different species. Apparently, the impact of some factors such as sex, gestation or lactation are not universal for all bat species [26]. However, in our study, we did not have enough samples from lactating bats for each species to evaluate species-specific differences. Overall, our study and others clearly show that high-risk periods for coronavirus shedding are related to age of bats and are highest when juvenile and subadults are present in large numbers in the colonies. Importantly, this period is specific for each bat species and can differ according to geographic sites and must therefore be evaluated for each species separately and in different areas to estimate spillover risk.

It is clear that humans are exposed to a wide diversity of bat coronaviruses in Africa, either directly through hunting and consumption or indirectly via contact with feces, urine or saliva on fruit or via guano. It will be important to evaluate to what extent some of these viruses can cross the species barrier or already cross the species barrier and lead to mild or asymptomatic infections. Serological assays with antigens from the different subgenera of alpha- and beta-CoVs need to be developed to evaluate this. Pre-existing immunity to other coronaviruses can also be one of the explanations for the lower morbidity of SARS-CoV-2 infections in Africa. This could be the case for coronaviruses that circulate at high rates in *Eidolon helvum,* which are one of the most common bats in Africa and roost in fruit trees in gardens of houses in cities and villages. For MERS-CoV, it is clear that multiple spill-over events occurred, but with limited subsequent spread in humans [57]. Although difficult to realize in practice given the mild symptoms and short viremic period, but given the high frequency of HCoV-H229E-related strains in bats, as observed in our and previous studies [38,58], molecular characterization of these or other mild coronaviruses in humans would be of interest to evaluate whether spillover events are still ongoing or whether the mild viruses, such as HCoV-229E, are the result of a single cross-species event.

Overall, we documented the high prevalence and genetic diversity of coronaviruses in different bat species from West and West Central Africa, especially in juveniles and subadults. More studies are needed to identify the high-risk periods, i.e., presence of high proportions of juvenile and subadult bats, to evaluate risk for spillover events for the different bat species. Importantly, our study shows that certain *Rhinolophus* colonies are highly infected with Sarbecoviruses and it can thus not completely be excluded that a spillover event could occur and lead to another SARS-CoV variant in humans. More cross-sectional and longitudinal studies are needed to document the extent of Sarbecovirus infections in *Rhinolophus* colonies in Africa to identify periods and environments in which spillover to humans is at higher risk. More studies on human behavior and diversity of interactions with bats are also needed in addition to viral characterization, as well as the development of specific antibody assays to evaluate past spillover events or to evaluate risk for future events. Finally, our study used only a small fragment in the RdRp region, which has been shown to be coherent with the classification of whole genome sequences for the large majority of strains, as shown by Wilkinson and colleagues [30]. Nevertheless, this classification has some limits, such as the absence of detection of recombinant lineages and provides no information on co-receptor use or factors associated with the evolution of these viruses in general [40,59]. Therefore, it is important to increase efforts to obtain more whole genome sequences from coronaviruses that circulate in bats in order to better document the diversity and evolution of these viruses in their natural hosts and their potential to infect humans.

## Figures and Tables

**Table 1:** Number of coronaviruses (CoVs) from the different subgenera of alpha- and beta-coronaviruses per bat genus/species. For the Nobecovirus (NobeCoV) subgenus, the different clades observed by phylogenetic analysis (Figure 5) are also indicated.

	AlphaCoronaviruses						BetaCoronaviruses								
	Unclass. CoV	DecaCoV	DuvinaCoV	MinunaCoV	PedaCoV	RhinaCoV	NobeCoV *Eidolon*	NobeCoV *Epomophorus*	NobeCoV *Lissonycteris*	NobeCoV *Rousettus (HKU9)*	NobeCoV *Rousettus*	NobeCoV *Megaloglossus*	HibeCoV	MerbeCoV	SarbeCoV
*Eidolon helvum*	2	-	-	-	-	-	233	3	-	-	-	-	-	-	-
*Epomophorus* sp. ^a^	2	-	-	-	-	-	2	50	-	1	-	-	-	-	-
*Epomops* sp. ^b^	-	-	-	-	-	-	-	5	-	-	-	-	-	-	-
*Hypsignathus monstrosus*	-	-	-	-	-	-	6	2	-	1	-	-	1	-	-
*Lissonycteris angolensis*	-	-	-	-	1	-	-	-	1	-	-	-	-	-	-
*Megaloglossus woermanni*	-	-	-	-	-	-	-	-	-	-	-	2	-	-	-
*Micropteropus pusillus*	-	-	-	-	-	-	-	5	-	-	-	-	-	-	-
*Myonycteris torquata*	-	-	-	-	-	-	-	-	6	-	-	-	-	-	-
*Nanonycteris* sp. ^c^	-	-	-	-	-	-	-	1	-	-	-	-	-	-	-
*Rousettus aegyptiacus*	5	16	-	-	-	-	3	-	-	64	8	-	1	-	-
**Total frugivorous**	**9**	**16**	**-**	**-**	**1**	**-**	**244**	**66**	**7**	**66**	**8**	**2**	**2**	**-**	**-**
*Coleura afra*	-	-	1	-	-	-	-	-	-	-	-	-	-	-	-
*Hipposideros* sp. ^c^	-	-	16	-	-	-	1	-	1	1	-	-	34	-	-
*Miniopterus* sp. ^c^	-	-	-	7	-	-	-	-	-	-	-	-	-	-	-
*Mops* sp. ^c^	-	-	-	3	-	-	3	-	-	-	-	-	-	-	-
*Mops/Chaerephon* sp. ^c^	-	-	-	-	-	-	2	2	-	-	-	-	-	-	-
*Nycteris* sp. ^c^	-	-	-	-	-	-	-	-	-	-	-	-	-	1	-
*Rhinolophus* sp. ^c^	54	-	-	1	-	67	5	-	-	2	1	-	31	-	114
**Total insectivorous**	**54**	**-**	**17**	**11**	**-**	**67**	**11**	**2**	**1**	**3**	**1**	**-**	**65**	**1**	**114**
**TOTAL**	**63**	**16**	**17**	**11**	**1**	**67**	**255**	**68**	**8**	**69**	**9**	**2**	**67**	**1**	**114**

^a^ Two *Epomophorus* species were observed: *E. gambianus* in Guinea, Cameroon and Western DRC and *E. labiatus* in Eastern DRC. ^b^ Two *Epomops* species were observed: *E. franqueti* in Cameroon and DRC and *E. buettikoferi.* ^c^ Identification at the species level was not possible for a significant proportion of samples tested and were therefore grouped at the genus level.

**Table 2:** Number of bats positive for coronaviruses (*n*+) on the total number of bats tested (N) and percentages of samples per sex and per bat genus/species.

	F	F	M	M	*p*−Values
	*n*+/N	% Pos	n+/N	% Pos	χ2 Test
**Frugivorous bats**					
Family *PTEROPODIDAE*					
*Casinycteris arginnis*	0/13	0.0	0/11	0.0	na ^d^
*Eidolon helvum*	122/335	36.4	170/404	42.1	0.0197 *
*Epomophorus* sp. ^a^	43/447	9.6	27/286	9.4	0.96, ns ^e^
*Epomops* sp. ^b^	3/155	1.9	5/102	4.9	na
*Hypsignathus monstrosus*	10/65	15.4	3/38	7.9	na
*Lissonycteris angolensis*	1/39	2.6	0/25	0.0	na
*Megaloglossus woermanni*	1/5	20.0	1/10	10.0	na
*Micropteropus pusillus*	2/80	2.5	3/85	3.5	na
*Myonycteris torquata*	2/38	5.3	4/53	7.5	na
*Nanonycteris* sp. ^c^	1/1	100	0/2	0.0	na
*Rousettus aegyptiacus*	68/472	14.4	46/319	14.4	0.99, ns
*Scotonycteris bergmansi*	0/1	0.0	-	-	na
**Subtotal frugivorous bats**	**243/1651**	**14.7**	**259/1335**	**19.4**	0.0007 *
**Insectivorous bats**					
Family *EMBALLONURIDAE*					
*Coleura afra*	1/1	100	-	-	na
Family *HIPPOSIDERIDAE*					
*Hipposideros* sp. ^c^	19/125	15.2	41/164	25.0	0.04 *
Family *MINIOPTERIDAE*					
*Miniopterus* sp. ^c^	2/2	100	6/6	100	na
Family *MOLOSSIDAE*					
*Chaerephon* sp. ^c^	0/65	0.0	0/51	0.0	na
*Mops* sp.	4/131	3.1	2/118	1.7	na
*Mops/Chaerephon* sp. ^c^	4/13	7.7	0/14	0.0	na
Family *NYCTERIDAE*					
*Nycteris* sp. ^c^	0/2	0.0	1/3	33.3	na
Family *RHINOLOPHIDAE*					
*Rhinolophus* sp. ^c^	174/485	35.9	130/411	31.6	0.2052, ns
Family *VESPERTILIONIDAE*					
*Myotis* sp. ^c^	0/2	0.0	0/2	0.0	na
*Scotophilus* sp. ^c^	0/3	0.0	-	-	na
**Subtotal insectivorous bats**	**204/829**	**24.6**	**180/769**	**23.4**	**0.57, ns**
**TOTAL**	**447/2480**	**18.0**	**439/2104**	**20.9**	0.0152 *

^a^ Two Epomophorus species were observed: *E. gambianus* in Guinea, Cameroon and Western DRC and *E. labiatus* in Eastern DRC. ^b^ Two Epomops species were observed: *E. franqueti* in Cameroon and DRC and *E. buettikoferi*. ^c^ Identification at the species level was not possible for a significant proportion of samples tested and were therefore grouped at the genus level. ^d^ na: not applicable (sample number is too low or does not exist). ^e^ ns non-significant *χ*^2^ test (*p*−value < 0.05). * significant *χ*^2^ test (*p*−value < 0.05).

**Table 3:** Number of bats positive for coronaviruses (*n*+) on total number (N) tested and percentages of positive (% pos) samples per bat genus/species positive per age category (adults and juveniles) and per species.

	Adult	Adult	Juvenile	Juvenile	*p*−Values
	*n*+/N	% Pos	*n*+/N	% Pos	χ^2^ Test
**Frugivorous bats**					
Family *PTEROPODIDAE*					
*Casinycteris arginnis*	0/24	0.0	na ^d^	na	na
*Eidolon helvum*	211/574	36.4	81/158	84.2	<10^−4^ *
*Epomophorus* sp. ^a^	76/693	10.9	2/37	5.4	na
*Epomops* sp. ^b^	8/250	3.2	0/6	0.0	na
*Hypsignathus monstrosus*	14/95	14.7	2/8	25	na
*Lissonycteris angolensis*	2/62	3.2	0/2	0.0	na
*Megaloglossus woermanni*	2/15	13.3	na	na	na
*Micropteropus pusillus*	5/160	3.1	0/2	0.0	na
*Myonycteris torquata*	6/86	6.7	na	na	na
*Nanonycteris* sp. *^c^*	1/3	33.3	na	na	na
*Rousettus aegyptiacus*	88/700	12.6	26/91	28.6	0.0001 *
*Scotonycteris bergmansi*	0/1	0.0	na	na	na
**Subtotal frugivorous bats**	**413/2667**	**15.5**	**111/304**	**36.5**	<10^−4^ *
**Insectivorous bats**					
Family *EMBALLONURIDAE*					
*Coleura afra*	1/1	100	na	na	na
Family *HIPPOSIDERIDAE*					
*Hipposideros* sp. ^c^	59/286	20.6	1/3	33.3	na
Family *MINIOPTERIDAE*					
*Miniopterus* sp. ^c^	8/8	100	na	na	na
Family *MOLOSSIDAE*					
*Chaerephon* sp. ^c^	0/89	0.0	0/25	0.0	na
*Mops* sp. ^c^	4/181	2.2	0/31	0.0	na
*Mops/Chaerephon* sp. ^c^	4/27	14.8	na	na	na
Family *NYCTERIDAE*					
*Nycteris* sp. *^c^*	1/5	20.0	na	na	na
Family *RHINOLOPHIDAE*					
*Rhinolophus* sp. *^c^*	283/868	32.6	21/28	75.0	<10^−4^ *
Family *VESPERTILIONIDAE*					
*Myotis* sp. *^c^*	0/4	0.0	na	na	na
*Scotophilus* sp. *^c^*	0/3	0.0	na	na	na
**Subtotal insectivorous bats**	**360/1472**	**24.5**	**22/87**	**25.3**	**0.9627, ns ^e^**
**Total**	**773/4139**	**18.7**	**133/391**	**34.0**	**<10^−4^**

^a^ Two Epomophorus species were observed: *E. gambianus* in Guinea, Cameroon and Western DRC and *E. labiatus* in Eastern DRC. ^b^ Two Epomops species were observed, *E. franqueti* in Cameroon and DRC and *E. buettikoferi*. ^c^ Identification at the species level was not possible for a significant proportion of samples tested and were therefore grouped at the genus level. ^d^ na: not applicable (sample number is too low or does not exist). ^e^ ns non-significant *χ*^2^ test (*p*−value < 0.05). * significant *χ*^2^ test (*p*−value < 0.05).

**Table 4:** Reproductive stage of female bats and coronavirus detection in bats from Cameroon. Number of bats positive (*n*+) on total number (N) tested and percentages of positive (%) for coronavirus per reproductive status.

Status	*n*+/N Tested	% Pos	*p*−Value
Adult females, gestation	11/47	23.4%	0.0882, ns
Adult females, no gestation	57/269	21.2%	
Adult females, lactation	10/85	11.8%	0.0349 *
Adult females, no lactation	93/410	22.7%	
Subadult females	150/389	38.6%	0.8833, ns
Juvenile females	58/146	39.7%	

* significant χ^2^ test (*p*−value < 0.05), ns: non-significant χ^2^ test (*p*−value < 0.05).

## Data Availability

Data are available upon request, and data on bat sampling are available on the EBO-SURSY website (https://rr-africa.woah.org/en/projects/ebo-sursy-en/, accessed on 20 November 2022). All new RdRp coronavirus sequences are available in GenBank with the following accession numbers: OP773878 to OP774616.

## Supplementary Materials

The following supporting information can be downloaded at: https://www.mdpi.com/article/10.3390/v15020337/s1, Supplementary Tables S1–S6: Table S1: Number of samples collected (N) at the different geographic locations in each country. The location of the different sites is visualized in Figure 1; Table S2: Number (*n*) and percentages (%) of bat genus/species positive for coronaviruses per country and per species; Table S3: Number (*n*) and proportion (%) of alpha and beta coronaviruses per bat genus/species, and *p*−values resulting from comparison of the proportions between alpha and betacoronavirus per genus/species, when applicable (Chi-2 test); Table S4: Number of bats positive for coronaviruses (*n* pos) on total number tested (N) and percentages (%) of samples per bat genus/species in Cameroon per age category (adults (A), immature adults (imm) and juveniles (J) and per species). The *p*−values result from comparison of the proportions of positive per genus/species, when applicable; Table S5: Genbank accession numbers and information on bat hosts for the new sequences obtained in this study; Table S6: Accession numbers and details of reference strains from alpha and betacoronavirus subgenera used in the phylogenetic tree in Figure 5.
