# Supplementary material for: Coronaviruses Are Abundant and Genetically Diverse in West and Central African Bats, including Viruses Closely Related to Human Coronaviruses
Source: Viruses. 2023 Jan 25;15(2):337. doi: 10.3390/v15020337 (PMC9967053; doi:10.3390/v15020337)
Supplement: Supplementary file 1 [file viruses-15-00337-s001.zip › Supplementary Table 1 to 4 revision_230118.pdf]

Supplementary Table S1 : Number of samples collected (N) at the different geographic locations in each country. The location of the different sites is visualized in **Figure 1**.

**Guinea**	**N**
Boffa	21
Boke	49
Conakry	175
Faranah	48
Forecariah	15
Gueckedou	71
Kankan	110
Kindia	224
Kissidougou	25
Koundara	249
Lola	16
Macenta	154
Mali	119
Maman/Mamou	164
Nzerekore	11
Siguri	58

**Cameroon**	**N**
Bipindi	1138
Campo	1
Doumo Pierre	41
Mambele	268
Mbankomo	56
Nkolbisson	62
Obala	131
Tibati	52
Yaoundé	462

**DRC**	
Beni	245
Bikoro	136
Butembo	33
Iboko	76
Ingende	44
Mangina	79
Mbandaka	284

Supplementary Table S2 : Number (n) and percentages (%) of bat genus/species positive for coronaviruses per country and per species.

	**DRC**		**Cameroon**		**Guinea**		**Total**	
	**n tested**	**n pos (%)**	**n tested**	**n pos (%)**	**n tested**	**n pos (%)**	**n tested**	**n (%)**
**Frugivorous bats**								
Pteropodidae								
Casinycteris arginnis	22	0 (0)	2	0 (0)	nt [d]	nt	24	0 (0)
Eidolon helvum	56	12 (21.4)	500	211 (42.2)	177	70 (39.5)	733	293 (39.9)
Epomophorus sp.[a]	258	9 (3.5)	nt	nt	479	63 (13.2)	737	72 (9.8)
Epomops sp. [b]	210	3 (1.4)	28	2 (7.1)	20	3 (15.0)	258	8 (3.1)
Hypsignathus monstrosus	8	0 (0)	76	9 (11.8)	19	3 (15.8)	103	12 (11.7)
Lissonycteris angolensis	10	0 (0)	9	0 (0)	46	2 (4.3)	65	2 (3.1)
Megaloglossus woermanni	12	2 (6.7)	3	0 (0)	nt	nt	15	2 (13.3)
Micropteropus pusillus	147	4 (2.7)	2	0 (0)	16	1 (6.3)	165	5 (3.0)
Myonycteris torquata	86	6 (7.1)	4	0 (0)	1	0 (0)	91	6 (6.6)
Nanonycteris sp. [c]	nt	nt	nt	nt	3	1 (33.3)	3	1 (33.3)
Rousettus aegyptiacus	nt	nt	226	49 (21.7)	565	65 (11.5)	791	114 (14.4)
Scotonycteris bergmansi	1	0 (0)	nt	nt	nt	nt	1	0 (0)
**subtotal**	**810**	**36 (4.4)**	**850**	**271 (31,9)**	**1326**	**208 (15.7)**	**2986**	**515 (17.2)**
**Insectivorous bats**								
Emballonuridae								
Coleura afra	nt	nt	1	1 (100)	nt	nt	1	1 (100)
Hipposideridae								
Hipposideros sp. [c]	47	0 (0)	206	51 (24.8)	38	10 (26.3)	291	61 (20.9)
Miniopteridae								
Miniopterus sp. [c]	nt	nt	9	9 (100)	nt	nt	9	9 (100)
Molossidae								
Chaerephon sp. [c]	3	0 (0)	nt	nt	9	0 (0)	12	0 (0)
Mops sp. [c]	21	0 (0)	235	6 (2.6)	nt	nt	256	6 (2.3)
Mops/Chaerephon sp. [c]	13	0 (0)	104	0 (0)	14	4 (28.6)	131	4 (3.1)
Nycteridae								
Nycteris sp. [c]	nt	nt	nt	nt	5	1 (20.0)	5	1 (20)
Rhinolophidae								
Rhinolophus sp.	nt	nt	786	289 (36.8)	113	17 (15.0)	899	306 (34.1)
Vespertilionidae								
Myotis sp. [c]	3	0 (0)	nt	nt	1	0 (0)	4	0 (0)
Scotophilus sp. [c]	nt	nt	nt	nt	3	0 (0)	3	0 (0)
**subtotal**	**87**	**0 (0)**	**1341**	**356 (26.5)**	**183**	**32 (17.5)**	**1611**	**388 (24.1)**
**Total**	**897**	**36 (4.0)**	**2191**	**627 (28.6)**	**1509**	**240 (15.9)**	**4597**	**903 (19.6)**

[a] Two *Epomophorus* species were observed, *E. gambianus* in Guinea, Cameroon and Western DRC and *E. labiatus* in Eastern DRC.

[b] Two *Epomops* species were observed, *E. franqueti* in Cameroon and DRC and *E. buettikoferi* in Guinea.

[c] Identification at species level was not possible for a significant proportion of samples tested and were therefore grouped at the genus level.

[d] nt: not tested

Supplementary Table S3: Number (n) and proportion (%) of alpha and beta coronaviruses per bat genus/species, and p-values resulting from comparison of the proportions between alpha and betacoronavirus per genus/species, when applicable (χ^2 test).

	Sequences n	alpha coronavirus n pos (%)	beta coronavirus n pos (%)	p-value χ2 test
**Frugivorous bats**				
Pteropodidae				
Eidolon helvum	238	2 (0.8)	236 (99.2)	$<10^{-4}$ *
Epomophorus sp. [a]	55	2 (3.6)	53 (96.4)	$<10^{-4}$ *
Epomops sp. [b]	5	0 (0.0)	5 (100)	na [d]
Hypsignathus monstrosus	10	0 (0.0)	10 (100)	na
Lissonycteris angolensis	2	1 (50.0)	1 (50.0)	na
Megaloglossus woermanni	2	0 (0.0)	2 (100)	na
Micropteropus pusillus	5	0 (0.0)	5 (100)	na
Myonycteris torquata	6	0 (0.0)	6 (100)	na
Nanonycteris sp. [c]	1	0 (0.0)	1 (100)	na
Rousettus aegyptiacus	97	21 (21.6)	76 (78.4)	$<10^{-4}$ *
**subtotal frugivorous bats**	**421**	**26 (6.2%)**	**395 (93.8%)**	**0.0029***
**Insectivorous bats**				
Emballonuridae				
Coleura afra	1	1 (100)	0 (0.0)	na
Hipposideridae				
Hipposideros sp. [c]	53	16 (30.2)	37 (69.8)	na
Miniopteridae				
Miniopterus sp. [c]	7	7 (100)	0 (0.0)	na
Molossidae				
Mops sp. [c]	6	3 (50.0)	3 (50.0)	na
Mops/Chaerephon sp. [c]	4	0 (0.0)	4 (100)	na
Nycteridae				
Nycteris sp. [c]	1	0 (0.0)	1 (100)	
Rhinolophidae				
Rhinolophus sp.[c]	275	122 (44.4)	153 (55.6)	0.0545, ns[e]
**subtotal insectivorous bats**	**347**	**149 (42.9%)**	**198 (57.1%)**	**0.44, ns**
**Total**	**768**	**175 (22.8)**	**593 (77.2)**	**$<10^{-4}$ ***

[a] Two E*pomophorus* species were observed, *E. gambianus* in Guinea, Cameroon and Western DRC and *E. labiatus* in Eastern DRC

[b] Two *Epomops* species were observed, *E. franqueti* in Cameroon and DRC and *E. buettikoferi*

[c] Identification at species level was not possible for a significant proportion of samples tested and were therefore grouped at the genus level.

[d] na: non applicable χ^2 test (numbers too low, or do not exist)

[e]ns : non significant χ^2 test (p-value>0.05)

* significant χ^2 test (p-value<0.05)

Supplementary Table S4: Number of bats positive for coronaviruses (n pos) on total number tested (N) and percentages (%) of samples per bat genus/species in Cameroon per age category (adults (A), immature adults (imm) and juveniles (J) and per species). The p-values result from comparison of the proportions of positive per genus/species, when applicable (χ^2 test).

	A n pos/N	A % pos	imm n pos/N	imm % pos	J n pos/N	J %pos	p-value
**Frugivorous bats**							
**Pteropodidae**							
Casinycteris arginnis	0/1	0.0	0/1	0.0	**-**	-	**na**[d]
Eidolon helvum	41/146	28.1	89/200	44.5	80/153	52.3	**ns**
Epomops franqueti	2/19	10.5	0/3	0.0	0/4	0.0	**ns**
Hypsignathus monstrosus	10/58	17.2	1/10	10	2/8	25.0	
Lissonycteris angolensis	0/6	0.0	0/2	0.0	0/1	0.0	**na**
Megaloglossus woermanni	0/2	0.0	0/1	0.0	-	-	**na**
Micropteropus pusillus	0/1	0.0	0/1	0.0	-	-	**na**
Myonycteris torquata	0/2	0.0	0/1	0.0	-	-	**na**
Rousettus aegyptiacus	5/109	4.6	18/38	47.4	26/79	32.9	**ns**
**Subtotal frugivorous bats**	**58/344**	**16.9**	**108/257**	**42**	**108/245**	**44.1**	**$<10^{-4}$ ***
**Insectivorous bats**							
Emballonuridae							
Coleura sp.	1/1	100	-	-	-	-	**na**
Hipposideridae							
Hipposideros sp.	27/108	25.0	23/97	23.7	1/1	100	**na**
Miniopteridae							
Miniopterus	3/3	100	5/5	100	-	-	**na**
Molossidae							
Chaerephon sp.	0/77	0.0	-	-	0/25	0.0	**na**
Mops sp.							
Rhinolophidae							
Rhinolophus sp.	117/411	28.5	150/347	43.2	21/26	80.7	**ns**
**Subtotal insectivorous bats**	**151/759**	**19.9**	**179/450**	**39.8**	**22/83**	**26.5**	**$<10^{-4}$ ***
**Total**	**209/1103**	**18.9**	**287/707**	**40.6**	**130/328**	**39.6**	**10^{-4} ***

[a] Two E*pomophorus* species were observed, *E. gambianus* in Guinea, Cameroon and Western DRC and *E. labiatus* in Eastern DRC

[b] Two *Epomops* species were observed, *E. franqueti* in Cameroon and DRC and *E. buettikoferi*

[c] Identification at species level was not possible for a significant proportion of samples tested and were therefore grouped at the genus level.

[d] na non applicable (numbers too low or do not exist)

[e]ns : non significant χ^2 test (p-value>0.05)

* significant χ^2 test (p-value<0.05)
